# Dystrophic Myopathy of the Diaphragm with Recurrent Severe Respiratory Failure is Congenital Myasthenic Syndrome 11

**DOI:** 10.3233/JND-221542

**Published:** 2023-03-07

**Authors:** J.J. Kramer, H.T.M. Boon, Q.H. Leijten, Henk Ter Laak, L. Eshuis, B. Kusters, J.L.M. van Doorn, E.J. Kamsteeg, B. Eymard, J. Doorduin, N.C. Voermans

**Affiliations:** a Department of Neurology, Donders Institute for Brain, Cognition and Behaviour, Radboud University Medical Centre, Nijmegen, The Netherlands; b Rijnstate Hospital, Arnhem, The Netherlands; c Department of Pathology, Radboud University Medical Centre, Nijmegen, The Netherlands; d Department of Genetics, Radboud University Medical Centre, Nijmegen, The Netherlands; e Institute de Myologie, Paris, France

**Keywords:** Congenital myasthenic syndrome, RAPSN, Rapsyn, CMS11, Whole exome sequencing, diaphragmatic dystrophy

## Abstract

We here present the case of a patient with a congenital myasthenic syndrome (CMS) due to pathogenic variants in the RAPSN gene. During childhood he experienced recurrent episodes of respiratory failure during respiratory infections. This and other cases were reported as isolated dystrophy of the diaphragmatic musculature. In adulthood, whole exome sequencing revealed two heterozygous pathogenic variants in the RAPSN gene. This led to the revision of the diagnosis to rapsyn CMS11 (OMIM:616326, MONDO:0014588). EMG, muscle ultrasound and the revision of muscle biopsies taken in childhood support this diagnosis. After the revision of the diagnosis, treatment with pyridostigmine was started. This resulted in a reduction of fatigability and an improvement in functional abilities and quality of life.

## INTRODUCTION

In 1993, our centre reported a boy with slight postnatal asphyxia related to isolated dystrophic diaphragmatic musculature [1]. Neonatal and early childhood development was complicated by multiple episodes of bronchopneumonia necessitating invasive ventilation. Multiple diagnostic tests were performed. Eventually, he was diagnosed with diaphragm muscular dystrophy. The authors referred to five similar cases of diaphragm muscular dystrophy, all of which had died in infancy [2–5].

In this report we present the 30-year follow-up of this patient and the diagnostic proceedings which have led to the diagnosis of congenital myasthenic syndrome (CMS) due to pathogenic variants in the RAPSN gene (NM_005055.5). CMS is a heterogenous group of genetic diseases that lead to a dysfunction of transmission of signals in the neuromuscular junction [6–9]. We additionally performed an EMG with repetitive nerve stimulation (RNS), ultrasound of the diaphragm and a revision of the biopsies. This report illustrates how this case and most likely other cases initially were classified before genetic testing became widely available and shows the long-term follow-up and treatment effect.

## CASE REPORT

A 28-year old man presented at the neurology outpatient department for analysis of increased fluctuating fatigable muscle weakness. He was born at 37 weeks by caesarian section after a pregnancy that was complicated by preeclampsia, breech position and decreased antenatal movements. Postnatally, he experienced a short period of hypoxia and generalized hypotonia. Physical examination showed mild resistance to passive movements of the elbows and hips. No muscular atrophy or muscle weakness was observed. A chest X-ray showed upwards displacement of the diaphragm on the right side, which was surgically corrected at 19 months with a diaphragm plication.

During the first two years of his life, he received physical therapy. He reached normal motor developmental milestones. Up to the age of six, he was admitted to the ICU eight times for respiratory weakness during episodes of bronchopneumonia. Each time, he was mechanically ventilated. After the age of six, he was hospitalized twice more with similar symptoms but without a need for ventilation.

Since early childhood he experienced fatigable muscle weakness, especially in periods of warm weather. Sports activities, such as gymnastics in school, posed difficulties. The fluctuating muscle weakness had been gradually progressive over the years.

Many diagnostic tests had been performed in early childhood. Laboratory tests of blood and CSF showed no abnormalities. Biochemical muscle tests showed lower oxidation speeds and decreased activity of some enzymes, though these results were inconclusive. Nerve conduction studies and electromyography were performed twice and were normal. The nerve conduction studies were performed without RNS. A neostigmine test was performed, which was negative.

**Fig. 2:**
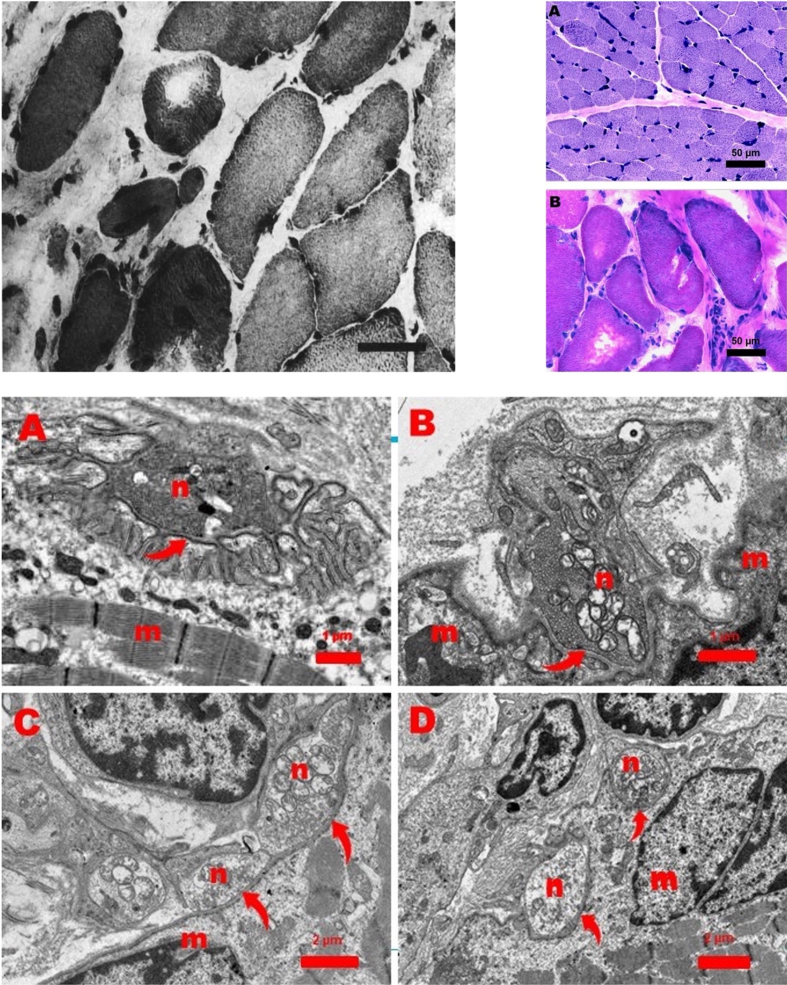
Top left: Original image of muscle biopsy of the diaphragm showing a dystrophic aspect with many hypertrophic muscle fibers in a matrix of connective tissue, as published in the report by Leijten et al. Top right: Muscle biopsy from the quadriceps muscle (A) and diaphragm muscle (B). Quadriceps muscle is normal. However, the diaphragm shows a dystrophic picture with hypertrophic fibers and increase of endomysial connective tissue. Bar: 50 micron. Hematoxilin-Phloxine (HP) stain. Bottom: Ultrastructure of a normal neuromuscular junction (NMJ) from a 11-month-old girl (Figure A). Figures B, C, and D show abnormal NJMs in the diaphragm of the patient in this report. Arrows point to the primary cleft between the neuronal (n) and muscular (m) part of the NMJs (Figure A, B, C, and D). The neuronal part (n) of the NMJs contains mitochondria and small vesicles with acetylcholine. Note the presence of many secondary clefts invaginating the muscular part (Figure A) and the almost complete absence of these invaginations in the abnormal NMJs.

In total, three muscle biopsies were performed. Two of these muscle biopsies were taken from the quadriceps muscle, one perioperatively from the diaphragm. The biopsies from the quadriceps muscle were performed at 17 days and 14 months after birth. They did not show any dystrophic characteristics, nor did immunohistochemical staining reveal any abnormalities. The biopsy of the diaphragm was taken during the surgical correction of the diaphragm (at 19 months). It showed a dystrophic aspect with many hypertrophic muscle fibers in a matrix of connective tissue. Most of these muscle fibers were type I muscle fibers, others resembled type IIC fibers. Some of these fibers contained round vacuoles filled with a basophilic substance. No fat cells or fiber splitting was observed (Fig. 2). Ultramicroscopic examination showed normal motor endplates. These findings led to the diagnosis of a dystrophy of the diaphragmatic musculature [1].

**Fig. 1:**
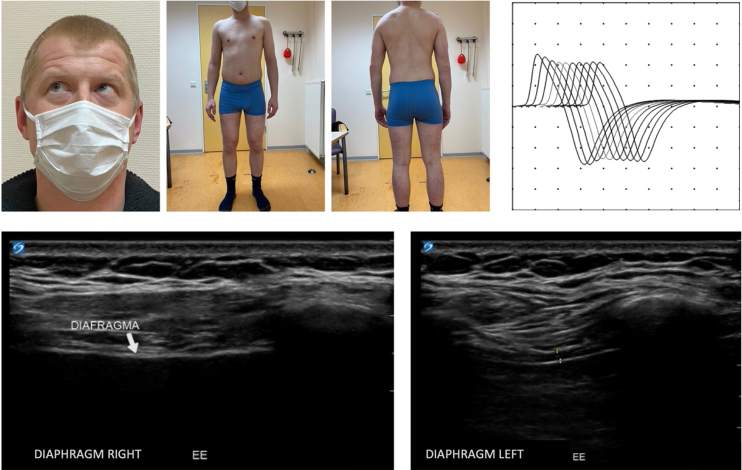
Top left: Clinical photographs, taken at the age of 32, showing a mild ophthalmoplegia of the right eye with upward-outward gaze and a lack of muscular atrophy. Top right: EMG with RNS showing decrement of CMAP amplitude of the trapezius muscle. Bottom: Ultrasound of right and left side of the diaphragm. The right side of the diaphragm shows severe muscle atrophy and no thickening during inspiration. The left side shows no abnormalities. The thickness at end-expiration is 1.7 mm and thickening ratio during inspiration is 3.1.

Neurological examination at the age of 28 showed mild limb-girdle muscle weakness (MRC 4), mild contractures of the knees and a mild ophthalmoplegia of the right eye with upward-outward gaze. No muscle atrophy was observed (Fig. 1). Whole exome sequencing revealed two variants in the *RAPSN* gene (c.264C>A p.(Asn88Lys) and c.737C>T p.(Ala246Val). The pathogenicity of the variants was established using ACMG/AMP guidelines for variant interpretation [10]. The p.(Asn88Lys) variant is considered pathogenic, because it is present in multiple patients (>30) with CMS11 in both the homozygous and compound heterozygous states, shows defective intracellular trafficking, and is not present in the homozygous state in large control populations, such as gnomAD (v2.1.1), despite its high allele frequency of 0.002579 in Europeans (Non-Finish) [11–13]. The p.(Ala246Val) variants was considered likely pathogenic, because it has been described in six other patients in the compound heterozygous state with the pathogenic p.(Asn88Lys) variant or with another likely pathogenic variant [12, 14–16]. It is extremely rare in controls (only four heterozygotes in gnomAD v.2.1.1 (246,778 alleles; allele frequency in non-Finish Europeans of 0.00005434). Segregation analysis showed that the parents are carriers, indicating that the patient is compound heterozygous for the two variants [11–13]. The patient (#00424068) and variants were submitted to the LOVD database [17]. EMG with RNS at the age of 33 showed a decrement of the compound muscle action potential amplitude of the trapezius muscle (Fig. 1). These findings confirm the genetic diagnosis of congenital myasthenic syndrome. The patient’s diaphragm and 16 limb and axial muscles were examined using (quantitative) muscle ultrasound. The ultrasound of the diaphragm showed severe muscle atrophy of the right hemidiaphragm (Fig. 1) and no thickening during inspiration. This is most likely the result of the corrective surgery of the diaphragm on the right side. The left hemidiaphragm did not show any abnormalities: thickness at end-expiration is 1.7 mm and thickening ratio during inspiration is 3.1. Quantitative muscle ultrasound showed no signs of atrophy and echo intensity was normal. Spirometry showed restrictive pulmonary function as a result of the paralysis of the right hemidiaphragm: FVC was 3.0 L (54% of predicted) with a normal FEV1/FVC of 86%.

Next, we reanalyzed the biopsy samples of both quadriceps biopsies and the diaphragm. Light microscopy of both quadriceps biopsies showed only minimal structural abnormalities. The first quadriceps biopsy showed type II predominance (82 %) and the range of muscle fiber diameters was smaller than normal, but the variability was not increased. The second quadriceps biopsy showed normal fiber diameters (Fig. 2), a slightly increased percentage of type I fibers, but no predominance.

In the diaphragm muscle, type I fibers were present. The exact percentage could not be calculated due to variable staining intensities with the ATPase stain, but oxidative enzyme stains suggest dominance of type I activity. Furthermore, diffuse infiltration of neutrophils and a clearly dystrophic aspect caused by endomysial fibrosis, many rounded hypertrophic fibers (Fig. 2), and sporadically an atrophic fiber were observed.

Electron microscopy of the diaphragm muscle revealed endomysial fibrosis, hypertrophic fibers, some fibers with core-like Z-line streaming central in the fiber, and occasionally a central region filled with myelin figures pointing to local necrosis. Furthermore, 5 abnormal neuromuscular junctions (NMJs) from 3 motor endplates were seen with severe loss of secondary synaptic clefts (Fig. 2).

After CMS was diagnosed, he started with pyridostigmine (up to 4 times 60 mg). This treatment proved to be successful, as the patient reported a reduction of the experienced muscle fatigability, an improvement in all daily activities and quality of life. No side effects of this treatment were reported. At the latest follow-up (age 33) he worked fulltime and was physically active in leisure activities (Table 1).

**Table 1:** Overview of course of including life events, treatments, and symptoms

	0–3 years	4–6 years	7–27 years	28–34 years
Life events	Initial diagnosis of isolated myopathy of the diaphragm	-	-	Diagnosis of CMS based on pathogenic variants in RAPSN gene
Treatment + side effects	Physical therapy Surgical correction of the diaphragm 5 ICU admissions due to respiratory insufficiency during bronchopneumonia	3 ICU admissions due to respiratory insufficiency during bronchopneumonia	2 admissions to hospital ward due to bronchopneumonia	Pyridostigmine treatment 60 mg four times per day. No side effects were reported
Motor milestone delay	-	-	-	-
Motor function UE	Contractures of elbows	-	-	Mild limb-girdle weakness
Motor function LE	Contractures of hips	-	-	Mild limb-girdle weakness Contractures of knees
Axial weakness	-	-	-	-
Axial stiffness	-	-	-	-
Urological dysfunction	-	-	-	-
Diplopia	-	-	-	-
Ptosis	-	-	-	-
Bulbar function	-	-	-	-
Sleep disturbances	-	-	-	Mild hypercapnia at night
Respiratory function	-	-	Restrictive respiratory function	Restrictive respiratory function
Fatigue	-	Easily fatigued, limited exercise tolerance	Easily fatigued, limited exercise tolerance	Easily fatigued, limited exercise tolerance (reduction of symptoms reported by patient since the start of pyridostigmine treatment)

“-“ = no information available, UE = upper extremities, LE = lower extremities, ICU = intensive care unit, CMS = congenital myasthenic syndrome.

## DISCUSSION

In this retrospective follow-up study of 30 years, we have reported the genetic diagnosis, the disease course and (non-)pharmacological treatments of a patient initially diagnosed with a dystrophic myopathy of the diaphragm. Genetic testing revealed two pathogenic variants of the RAPSN gene, compatible with the diagnosis of CMS type 11. The findings of physical examination, EMG with RNS of the trapezius muscle, and reevaluation of the muscle biopsy confirmed this diagnosis.

The recurrent episodes of severe respiratory weakness during pulmonary infections in infancy and early childhood are typical for rapsyn CMS [6, 7, 9, 18]. A possible explanation for these respiratory crises becoming less frequent with age is the maturing of the immune system with age. More immunity for respiratory pathogens is acquired as a person ages. Furthermore, young children are more likely to pass on pathogens to each other due to their social behavior [19, 20]. The respiratory weakness experienced at birth, generalized hypotonia, contractures of the elbows and hips, and the positive response to pyridostigmine treatment are also commonly reported in patients with RAPSN CMS. Ophthalmoplegia is less common. Bulbar symptoms, motor deficit and finger contractures, typical characteristics of RAPSN CMS, were not observed in this patient [6–8].

Muscle biopsies are often abnormal in CMS, including type I fiber dominance, increased fiber size variation, and cores or minicores and mild local endomysial fibrosis [11, 16, 21–26]. The patient in this report showed some variation of fiber size in the first quadriceps biopsy, but no type I fiber dominance. However, the light microscopy of the diaphragm biopsy was very abnormal, resulting in the initial diagnosis of dystrophic myopathy of the diaphragm.

Ultrastructural investigations of NMJs in CMS11 are limited to 10 patients, all of which were reported to contain abnormal NMJs with severe to almost complete disappearance of the secondary clefts [11, 24–26]. Our observations of the NMJ ultrastructure are in agreement with these findings. The finding of an isolated normal NMJ [24] may explain why endplates were reported as normal in the first publication of our patient.

The abnormal NMJ structure is considered to be a direct consequence of the function of rapsyn: it binds and anchors the acetylcholine receptor (AChR) to the postsynaptic membrane. In accord, it interacts with the AChR and a range of proteins that regulate the cytoskeleton. Rapsyn also interacts with signaling molecules. Recent studies show that it possesses E3 ligase activity that is required for NMJ formation, revealing a novel function of this classic adaptor protein. Identifying rapsyn as a signaling molecule [27, 28]. This has increased the understanding of the mechanisms of NMJ formation and maintenance.

Equally severe changes in the diaphragm muscle could not be found in literature on CMS11. Furthermore, these changes contrast to the changes in both quadriceps biopsies. The diaphragm muscle is clearly dystrophic with hypertrophic fibers and severe fibrosis. This may be explained by the continuous strong activity of the diaphragm muscle needed for respiration. This condition may be compared to so-called excessive exercise training. This type of training may result in fiber hypertrophy, muscle damage and neutrophil infiltration, fiber regeneration, and endomysial fibrosis [29, 30].

We here added muscle ultrasound of the diaphragm and limb and axial muscles. We found right hemidiaphragm paralysis as a result of the corrective surgery in early childhood resulting in a restrictive lung function. There were no abnormalities in quantitative muscle ultrasound of the limb and axial muscles. There is no literature available on quantitative muscle ultrasound in CMS due to pathogenic variants of the RAPSN gene. However, muscle magnetic resonance imaging (MRI) showed mixed findings of the degree of muscle fat infiltration in three patients with these pathogenic variants in the RAPSN gene, ranging from normal to marked [31].

In the report on this case, Leijten et al. referred to a number of similar cases of children with an isolated myopathy of the diaphragm. All these children had passed away in the neonatal period. One of these reports was by Bergen et al. [2]. They described a girl who, shortly after birth, experienced respiratory distress and generalized hypotonia. A chest X-ray and stimulation of the phrenic nerve showed there was no movement of the right diaphragm. She died during an episode of pneumonia at the age of three months. The findings in this case report are similar to the findings in the patient in our centre, which might point to a diagnosis of (rapsyn) CMS in her.

Other patients with the same pathogenic variants as the patient in our report have been described in literature. In their paper, Miler et al. describe a patient whose phenotype is like that of our patient, presenting at birth with arthrogryposis and requiring ICU admission in childhood during a varicella infection. Like the patient in our report, this patient also showed a positive response to treatment with pyridostigmine [26].

Congenital myasthenic syndromes are rare. One study showed a prevalence of 9.2/1,000,000 in children in the UK [32]. It is likely that this is an underestimation because CMS may manifest only with mild symptoms or may be overlooked by clinicians due to a lack of knowledge on the subject. It is estimated that about 20% of all CMS are caused by pathogenic variants in the RAPSN gene [7]. The RAPSN c.264C>A variant is a highly prevalent variant in the Western and Central European population [8]. Furthermore, many adult patients with CMS have received other diagnoses in the pre-genetic era. It is therefore likely that there are many more people with CMS11 that have gone undiagnosed. Correct diagnosis is crucial because many CMS subtypes respond well to treatment with pyridostigmine or other drugs. There should be a lower threshold for genetic testing in patients with symptoms of CMS. Patients with symptoms of CMS who received an alternative diagnosis in the pre-genetic era may be re-analysed. We expect that early diagnosis enabled by next generation sequencing and adequate treatment leads to a significant improvement in the quality of life of these patients.

## References

[1] Leyten QH , et al. Dystrophic myopathy of the diaphragm in a neonate with severe respiratory failure during infectious episodes. Neuromuscul Disord. 1993;3(1):51–5. 8329889 10.1016/0960-8966(93)90041-h

[2] Bergen BJ , Sangalang VE , Aterman K . Isolated myopathic involvement of the diaphragmatic musculature in a neonate. Ann Neurol. 1977;1(4):403–7. 617257 10.1002/ana.410010414

[3] Bosman C , et al. Diaphragmatic paralysis due to partial diaphragmatic hypoplasia mimicking a localized muscular dystrophy: A case report. Clin Neuropathol. 1988;7(1):33–8. 3286072

[4] De Reuck J , et al. A progressive congenital myopathy. Initial involvement of the diaphragm with type I muscle fiber atrophy. Eur Neurol. 1977;15(4):217–56. 872842 10.1159/000114836

[5] Lewis AJ , Besant DF . Muscular dystrophy in infancy: Report of 2 cases in siblings with diaphragmatic weakness. The Journal of Pediatrics. 1962;60(3):376–84. 14464836 10.1016/s0022-3476(62)80063-8

[6] Engel AG , et al. Congenital myasthenic syndromes: Pathogenesis, diagnosis, and treatment. Lancet Neurol. 2015;14(4):420–34. 25792100 10.1016/S1474-4422(14)70201-7 PMC4520251

[7] Finlayson S , Beeson D , Palace J . Congenital myasthenic syndromes: An update. Pract Neurol. 2013;13(2):80–91. 23468559 10.1136/practneurol-2012-000404

[8] Finsterer J . Congenital myasthenic syndromes. Orphanet J Rare Dis. 2019;14(1):57. 30808424 10.1186/s13023-019-1025-5 PMC6390566

[9] Hantaï D , Nicole S , Eymard B . Congenital myasthenic syndromes: An update. Curr Opin Neurol. 2013;26(5):561–8. 23995276 10.1097/WCO.0b013e328364dc0f

[10] Richards S , et al. Standards and guidelines for the interpretation of sequence variants: A joint consensus recommendation of the American College of Medical Genetics and Genomics and the Association for Molecular Pathology. Genet Med. 2015;17(5):405–24. 25741868 10.1038/gim.2015.30 PMC4544753

[11] Ohno K , et al. Rapsyn mutations in humans cause endplate acetylcholine-receptor deficiency and myasthenic syndrome. Am J Hum Genet. 2002;70(4):875–85. 11791205 10.1086/339465 PMC379116

[12] Müller JS , et al. Rapsyn N88K is a frequent cause of congenital myasthenic syndromes in European patients. Neurology. 2003;60(11):1805–10. 12796535 10.1212/01.wnl.0000072262.14931.80

[13] Cossins J , et al. Diverse molecular mechanisms involved in AChR deficiency due to rapsyn mutations. Brain. 2006;129(Pt 10):2773–83. 16945936 10.1093/brain/awl219

[14] Westra D , et al. Panel-based exome sequencing for neuromuscular disorders as a diagnostic service. J Neuromuscul Dis. 2019;6(2):241–58. 31127727 10.3233/JND-180376

[15] Abicht A , et al. Congenital myasthenic syndromes: Achievements and limitations of phenotype-guided gene-after-gene sequencing in diagnostic practice: A study of 680 patients. Hum Mutat. 2012;33(10):1474–84. 22678886 10.1002/humu.22130

[16] Saito M , et al. Successful treatment of congenital myasthenic syndrome caused by a novel compound heterozygous variant in RAPSN. Brain Dev. 2022;44(1):50–5. 34565654 10.1016/j.braindev.2021.09.001

[17] Fokkema IF , et al. LOVD v.2.0: The next generation in gene variant databases. Hum Mutat. 2011;32(5):557–63. 21520333 10.1002/humu.21438

[18] Natera-de Benito D , et al. Long-term follow-up in patients with congenital myasthenic syndrome due to RAPSN mutations. Neuromuscul Disord. 2016;26(2):153–9. 26782015 10.1016/j.nmd.2015.10.013

[19] Reina J , Cabrerizo M . Age distribution of acute respiratory infections caused by enteroviruses in the child population. Enferm Infecc Microbiol Clin. 2017;35(9):608–9. 28104308 10.1016/j.eimc.2016.12.007

[20] Chen Y , Kirk MD . Incidence of acute respiratory infections in Australia. Epidemiol Infect. 2014;142(7):1355–61. 24103382 10.1017/S0950268813002471 PMC9151186

[21] Radke J , et al. The curse of apneic spells. Semin Pediatr Neurol. 2018;26:56–8. 29961520 10.1016/j.spen.2017.03.006

[22] Ioos C , et al. Congenital myasthenic syndrome due to rapsyn deficiency: Three cases with arthrogryposis and bulbar symptoms. Neuropediatrics. 2004;35(4):246–9. 15328566 10.1055/s-2004-820993

[23] Das AS , Agamanolis DP , Cohen BH . Use of next-generation sequencing as a diagnostic tool for congenital myasthenic syndrome. Pediatr Neurol. 2014;51(5):717–20. 25194721 10.1016/j.pediatrneurol.2014.07.032

[24] Yasaki E , et al. Electrophysiological and morphological characterization of a case of autosomal recessive congenital myasthenic syndrome with acetylcholine receptor deficiency due to a N88K rapsyn homozygous mutation. Neuromuscul Disord. 2004;14(1):24–32. 14659409 10.1016/j.nmd.2003.07.002

[25] Maselli RA , et al. Rapsyn mutations in myasthenic syndrome due to impaired receptor clustering. Muscle Nerve. 2003;28(3):293–301. 12929188 10.1002/mus.10433

[26] Banwell BL , et al. Novel truncating RAPSN mutations causing congenital myasthenic syndrome responsive to 3,4-diaminopyridine. Neuromuscul Disord. 2004;14(3):202–7. 15036330 10.1016/j.nmd.2003.11.004

[27] Xing G , et al. A mechanism in agrin signaling revealed by a prevalent Rapsyn mutation in congenital myasthenic syndrome. Elife. 2019;8. 10.7554/eLife.49180 PMC6779466 31549961

[28] Xing G , Xiong WC , Mei L . Rapsyn as a signaling and scaffolding molecule in neuromuscular junction formation and maintenasnce. Neurosci Lett. 2020;731:135013. 32344108 10.1016/j.neulet.2020.135013

[29] Schneider BS , Tiidus PM . Neutrophil infiltration in exercise-injured skeletal muscle: How do we resolve the controversy? Sports Med 2007;37(10):837–56. 17887810 10.2165/00007256-200737100-00002

[30] Pessina P , et al. Novel and optimized strategies for inducing fibrosis in vivo: Focus on Duchenne Muscular Dystrophy. Skelet Muscle. 2014;4:7. 25157321 10.1186/2044-5040-4-7 PMC4142391

[31] Finlayson S , et al. Muscle magnetic resonance imaging in congenital myasthenic syndromes. Muscle Nerve. 2016;54(2):211–9. 26789134 10.1002/mus.25035 PMC4982021

[32] Parr JR , et al. How common is childhood myasthenia? The UK incidence and prevalence of autoimmune and congenital myasthenia. Arch Dis Child. 2014;99(6):539–42. 24500997 10.1136/archdischild-2013-304788

